# Trends in scientific research in *Insights into Imaging*: a bibliometric review

**DOI:** 10.1186/s13244-019-0766-y

**Published:** 2019-08-28

**Authors:** Juan Carlos Valderrama-Zurián, Lourdes Castelló-Cogollos, Rafael Aleixandre-Benavent

**Affiliations:** 1 Instituto de Documentación y Tecnologías de la Información (INDOTEI), Universidad Católica de Valencia San Vicente Mártir, Valencia, Spain; 2 UISYS, Joint Research Unit, Universitat de València-CSIC, Palacio Cerveró, Plaza Cisneros, 4, 46003 Valencia, Spain; 3 Departament de Sociologia i Antropologia Social, Universitat de València, Valencia, Spain; 4 Instituto de Gestión de la Innovación y del Conocimiento-Ingenio (CSIC-Universitat Politècnica de València), Palacio Cerveró, Plaza Cisneros, 4, 46003 Valencia, Spain

**Keywords:** Publications, Bibliometrics, Databases, Bibliographic, Journal impact factor, Diagnostic imaging

## Abstract

**Objectives:**

To analyse the coverage and main bibliometric indicators of *Insights into Imaging* in Scopus and the Emerging Sources Citation Index (ESCI) from the Web of Science Core Collection database.

**Methods:**

The evolution of journal production in the Scopus database was analysed according to document types, collaboration indexes between authors and institutions, and citation indicators (number of citations, SCImago Journal Rank, quartile, h-index, and most cited works). Networks of collaboration among authors, institutions, and countries were also analysed, as well as those of co-word networks. As a complementary source of information, the Emerging Source Citation Index from the Web of Science database was also considered.

**Results:**

Four hundred seventy-four papers were included in Scopus and 292 in ESCI. The index of collaboration was 5.18 for authors and 2.74 for institutions. International collaboration occurred in 24.7% of the papers. The number of citations received in Scopus (4295) exceeds the number of citations received in ESCI (1177). The average number of citations per paper was 9.06 in Scopus versus 4.03 in ESCI. The h-index was 29 in Scopus and 16 in ESCI. Several collaborative groups were identified at the national and international level.

**Conclusions:**

There is a progression of *Insights into Imaging* in the ranking of journals in the area that, if maintained, will allow it to remain in the first quartile in the Scopus database. The main topics of interest were technologies such as ‘Magnetic resonance imaging’, ‘Computed tomography’, ‘Radiology’, and ‘Ultrasonography’ and diseases such as ‘Breast cancer’ and those related to ‘Paediatrics’.

## Key points

- Increasing trend of *Insights into Imaging* in the ranking of journals.
- Social network analysis identified the main groups of research.
- Collaboration among European countries, USA, and Australia predominated.
- The average citation/work is higher than other journals in the area.
- Nine of the most cited papers have MRI as their central topic.

## Introduction

*Insights into Imaging* is a peer-reviewed journal founded in 2010 and published by the European Society of Radiology (ESR). The journal is edited under the brand SpringerOpen. The journal is the official journal of the ESR and includes on its editorial board representatives from eleven scientific societies that embrace several areas of the biomedical sciences. It is considered the world over as a high-quality and up-to-date source of information in the field of radiology [1].

Advances in the study of diseases through diagnostic imaging have been considerable in recent decades, as evidenced by the number of articles published in PubMed/Medline (https://www.ncbi.nlm.nih.gov/pubmed/), which according to the MeSH (‘Diagnostic Imaging’[Mesh]) has gone from 51,068 in 2001 to almost 100,000 in recent years. A better understanding of the evolution of the bibliometric indicators of *Insights into Imaging* and an awareness of their complexities and challenges can contribute to improving their future development.

Some studies have analysed the scientific activity of global diagnostic imaging [2–11]. However, little is known about the evolution of the *Insights into Imaging* indicators in the two international databases that provide citation and impact data and the indicators in which it is indexed, Scopus and the Emerging Sources Citation Index of the Web of Science Core Collection (hereinafter, ESCI).

### Given this background, our goal is threefold

- To analyse the evolution of the main scientometric indicators of production, collaboration, and impact of *Insights into Imaging* in the ESCI and Scopus databases during the years in which these databases have indexed the journal.
- To provide reliable data on the actual coverage of these sources and warn about how the different editorial policies of the databases related to the indexing of the records can produce variable and source-dependent indicators, due to discrepancies between the data provided by the databases that process the records and the citations.
- To identify groups of more active authors, institutions, and countries that are considered at the research forefront in the area covered by the journal, as well as the publication of articles in *Insights into Imaging* by the members of the editorial board.
- To determine the most relevant topics and research trends from the analysis of the keywords assigned to the documents.

## Materials and methods

The methodology consisted of several phases:

- Search, which comprises the following: first, downloading of records from the Scopus database, and second, the standardisation of data of authors, institutions, and keywords, which consisted of unifying the different variants of the name of the same author or institution and grouping synonymous keywords, in addition to identifying authors belonging to the editorial board.
- Variables, which include the following: first, the calculation of production indicators: number of articles classified by type of work and year of publication; production by authors, institutions, and countries; and number of papers published by editorial board members; second, the calculation of collaboration indicators (annual evolution of the collaboration index among authors, institutions, and countries; collaboration networks among authors, institutions, and countries based on papers included in Scopus); and third, indicators based on citations (number of citations, average citations per paper, SCImago Journal Rank (SJR), position of the journal in the world ranking of radiology, h-index, most-cited papers (hot papers)).
- Databases. Three sources were used to obtain these data and indicators: the Scopus database, SCImago Journal & Country Rank, and the Emerging Sources Citation index from the Web of Science Core Collection database. All these indicators are defined in the aforementioned sources and were also described and applied in previous works [12–18], except for the index of citations per year of the most-cited works, which is the result of dividing the number of citations by the years elapsed since the publication of the study. Data on citations correspond to January 19, 2019.

## Results

### General data: annual evolution and document typology

**Fig. 1:**
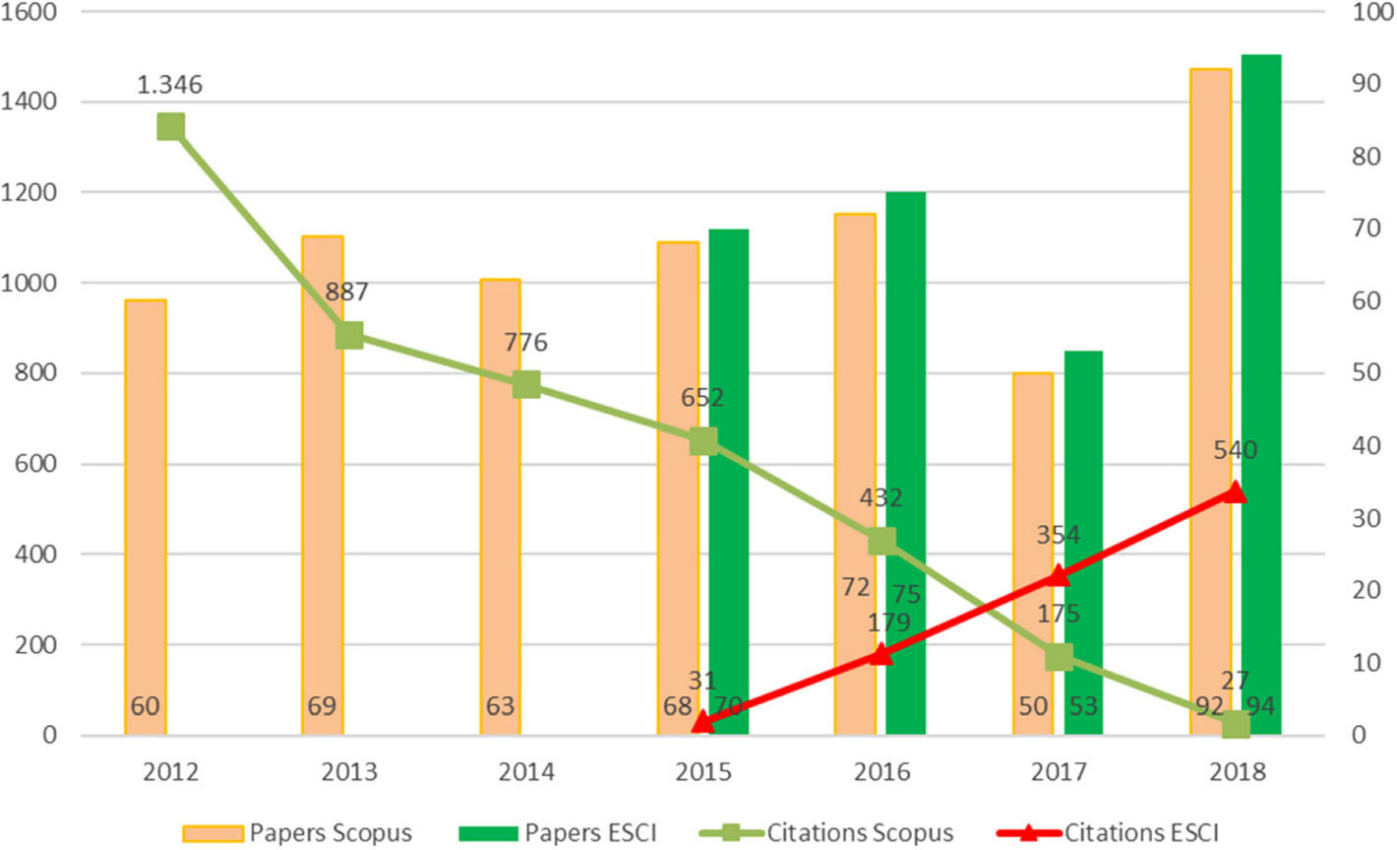
Annual evolution of published papers and citations in Scopus and ESCI databases

The annual evolution of the journal’s coverage in Scopus (since 2012) and ESCI (since 2015) is shown in Fig. 1. Scopus included 474 papers while ESCI included 292. As for document coverage, some discrepancies between the two databases can be seen. The percentage of the document type ‘articles’ coincided in both databases (24.7%), but the percentage of the document type ‘revisions’ was somewhat higher in ESCI than in Scopus (72% as opposed to 68.3%, respectively), while the percentage of editorials was higher in Scopus than in ESCI (2.3% versus 0.7%, respectively).

### Scientific production of authors, institutions, and countries

One thousand nine hundred fifteen different authors published 474 papers. As mentioned above, data on the production and collaboration of authors, institutions, and countries have been extracted from the registers included in Scopus. Table 1 presents the 23 authors who published 5 or more papers. Three authors stand out with more than 10 papers: Tonolini (*n* = 20; ‘Luigi Sacco’ University Hospital, Milan, Italy), Chaturvedy (*n* = 18; University of Rochester, New York, USA), and Parizel (*n* = 11; Antwerp University Hospital, Belgium). Seventeen papers were signed by the European Society of Radiology. The highest number of citations received was for Puderbach (*n* = 280, but only 4 published papers), followed by Wildberger (*n* = 125) and two authors with 116 citations: Beets, and Tan and Lobbes (with only 4 published papers). The citation index per article is also higher for these authors.

**Table 1:** Most productive authors (*n* > 4 papers)

Author	Papers	Citations	Citations/paper
Tonolini, M	20	56	2.80
Chaturvedi, A	18	52	2.89
European Society of Radiology (ESR)	17	101	5.94
Parizel, PM	11	85	7.73
Rajiah, P	8	45	5.63
Bianco, R	8	37	4.63
Ierardi, AM	7	9	1.29
Carrafiello, G	7	8	1.14
Wildberger, JE	6	125	20.83
Ettorre, GC	6	62	10.33
Palmucci, S	6	62	10.33
Sardanelli, F	6	31	5.17
Becker, M	6	92	15.33
Vanhoenacker, FM	5	38	7.60
Guermazi, A	5	74	14.80
Foti, PV	5	46	9.20
Ippolito, S	5	18	3.60
Schieda, N	5	71	14.20
Sconfienza, LM	5	39	7.80
Nyhsen, CM	5	46	9.20
Adam, EJ	5	46	9.20
Beets Tan, RGH	5	116	23.20
Roemer, FW	5	83	16.60

As for the participation of editorial board members, 23 of its 49 members (46.9%) published 56 papers (11.8% of the total number of papers published by the journal).

There were 41 institutions with 5 or more papers out of a total of 747 papers, and they are presented in Table 2. The institution that published the highest number of papers was the European Society of Radiology (*n* = 25), followed by ‘Luigi Sacco’ University Hospital (Milan, Italy) (*n* = 20), University of Rochester (New York, USA) (*n* = 19), and Antwerp University Hospital (Belgium) (*n* = 14). The number of citations received was higher for Guy’s and St Thomas’ Hospital (UK) (*n* = 401 citations), followed by University Hospital Heidelberg (Germany) (*n* = 299) and German Cancer Research Center (Germany) (*n* = 280), both with 5 published papers. The citations per paper ranking is led by these three institutions but in another order: University Hospital Heidelberg (C/*P* = 59.8), followed by German Cancer Research Center (C/*P* = 56) and Guy’s and St Thomas’ Hospital (C/*P* = 44.56).

**Table 2:** Most productive institutions (*n* > 5 published papers)

Institutions	Countries	Papers	Citations	Citations/paper
European Society of Radiology (ESR)	Austria	25	141	5.64
Luigi Sacco University Hospital	Italy	20	56	2.80
University of Rochester	USA	19	43	2.26
Antwerp University Hospital	Belgium	14	162	11.57
Università degli Studi di Milano	Italy	12	65	5.42
University of Antwerp	Belgium	11	87	7.91
Geneva University Hospital	Switzerland	10	93	9.30
Università di Roma Sapienza	Italy	10	80	8.00
Maastricht University	The Netherlands	10	171	17.10
IRCCS Policlinico San Donato	Italy	9	100	11.11
Guy’s and St. Tomas Hospital	UK	9	401	44.56
Ghent University Hospital	Belgium	9	120	13.33
The Ottawa Hospital	Canada	9	98	10.89
University of Toronto	Canada	8	58	7.25
University of Ottawa	Canada	8	85	10.63
Lund University	Sweden	7	56	8.00
University of Verona	Italy	6	28	4.67
University Hospital Zurich	Switzerland	6	91	15.17
Centre Hospitalier de l’Université de Montréal (CHUM)	Canada	6	50	8.33
Hospital Clinic de Barcelona	Spain	6	64	10.67
Beth Israel Deaconess Medical Center	USA	6	74	12.33
Boston University	USA	6	86	14.33
University of Geneva	Switzerland	6	81	13.50
Skåne University Hospital	Sweden	6	46	7.67
University of Pisa	Italy	6	45	7.50
Great Ormond Street Hospital	UK	6	68	11.33
University Hospital Policlinico-Vittorio Emanuele	Italy	6	62	10.33

**Fig. 2:**
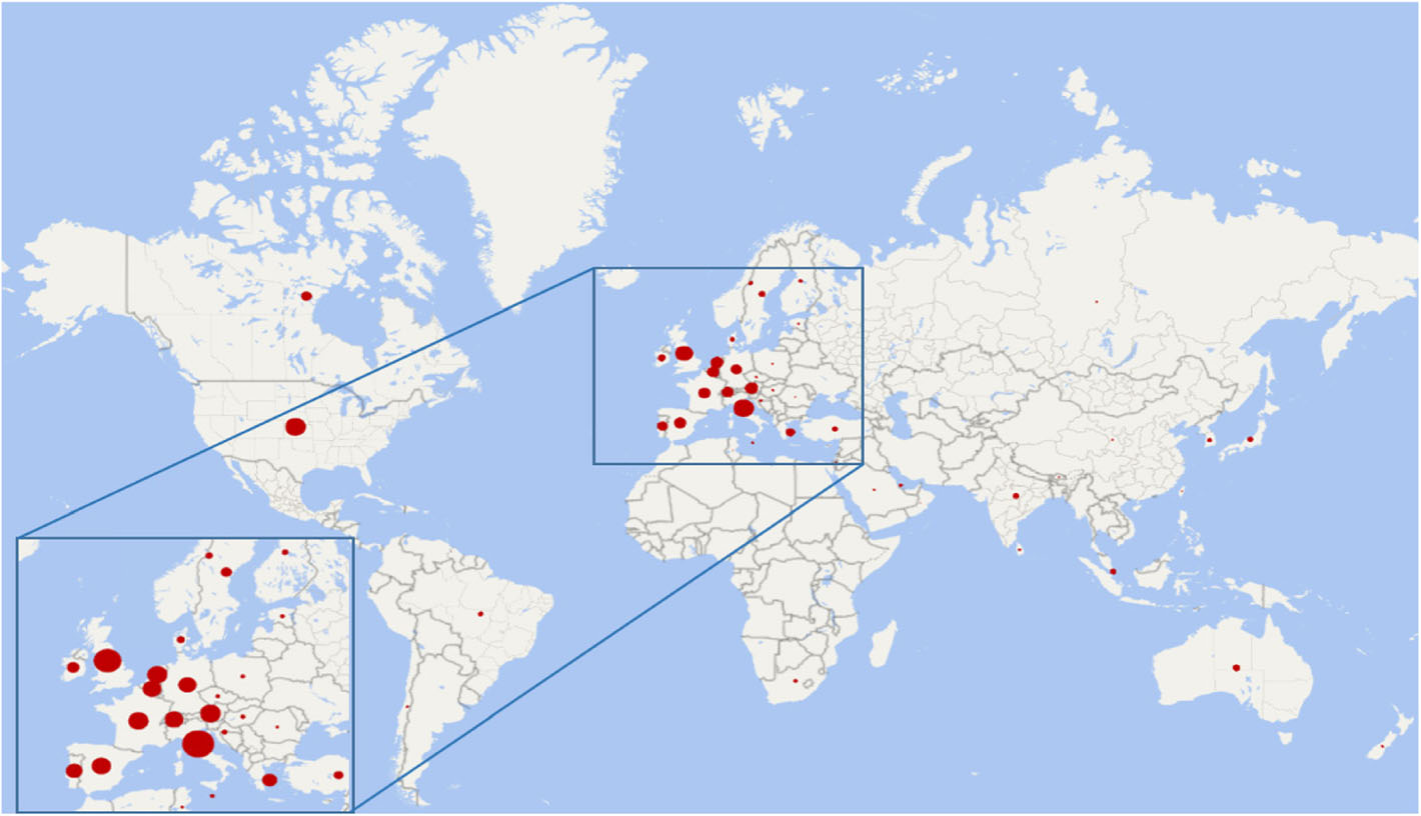
World map of countries’ production

The production by country was led by Italy (*n* = 92, 19.4%), followed by the USA (*n* = 88, 18.6%), the UK (*n* = 68, 14.3%), Austria (*n* = 38, 8%), the Netherlands (*n* = 37, 7.8%), France (*n* = 36, 7.6%), Spain (*n* = 34, 7.2%), Belgium (*n* = 32, 6.8%), and Switzerland (*n* = 31, 6.5%). As seen, European countries published most of the works (Fig. 2).

### Patterns of collaboration

#### Collaboration among authors

**Fig. 3:**
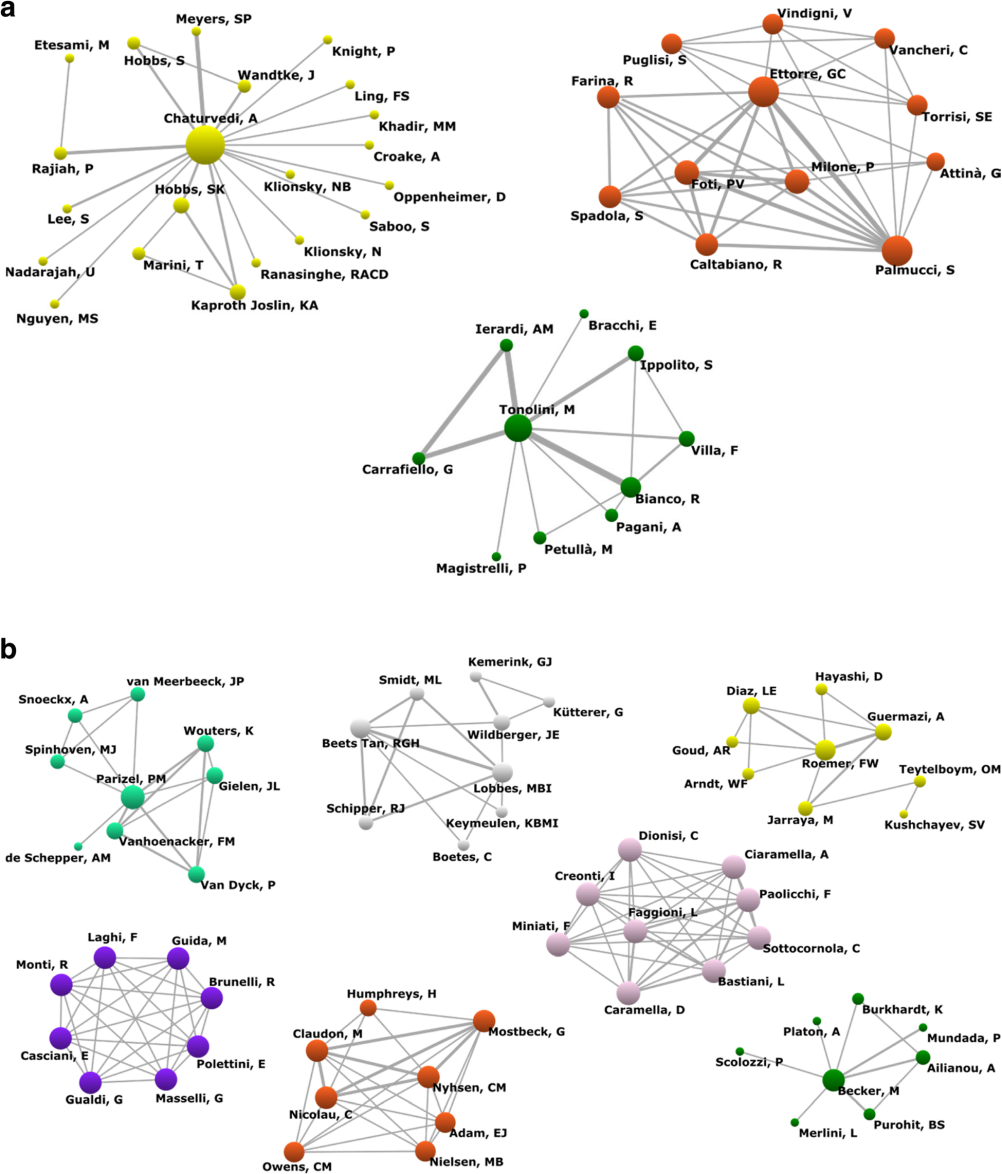
Network of collaboration between authors. **a** Groups of 21, 12, and 10 authors. **b** Groups with 9 or 8 members (source: Scopus database)

The authors’ collaboration index during the whole period was 5.18 authors per work. The analysis of social networks made it possible to identify 56 groups in which 278 authors who had published at least one collaborative work participated. Figure 3a shows the groups with the highest number of members: a group of 21 authors, one of 12, and one of 10. Figure 3b shows four groups with 9 members and four groups with 8 members.

The group with the highest number of authors has Chaturvedi as its main author, and Chaturvedi is affiliated with the University of Rochester (New York, USA). The second group has as central authors Ettore and Palmucci, who work at the University Hospital ‘Policlinico-Vittorio Emanuele’ (Catania, Italy). In the third group, Tonolini is the central author and belongs to the ‘Luigi Sacco’ University Hospital (Milan, Italy). The four groups of 9 members in the upper part of Fig. 3b belong to institutions in Belgium (Antwerp University Hospital), the Netherlands (The Netherlands Cancer and Maastricht University), Italy (National Research Council), and Germany (University of Erlangen-Nuremberg). Lastly, of the three groups of 8 authors, the one on the left side of the figure includes researchers from Italy (Sapienza Università di Roma); the group in the centre is international and includes researchers from the UK, Spain, Austria, France, and Ireland; and the group on the right side includes researchers from Switzerland (Geneva University Hospitals).

#### Collaboration among institutions

**Fig. 4:**
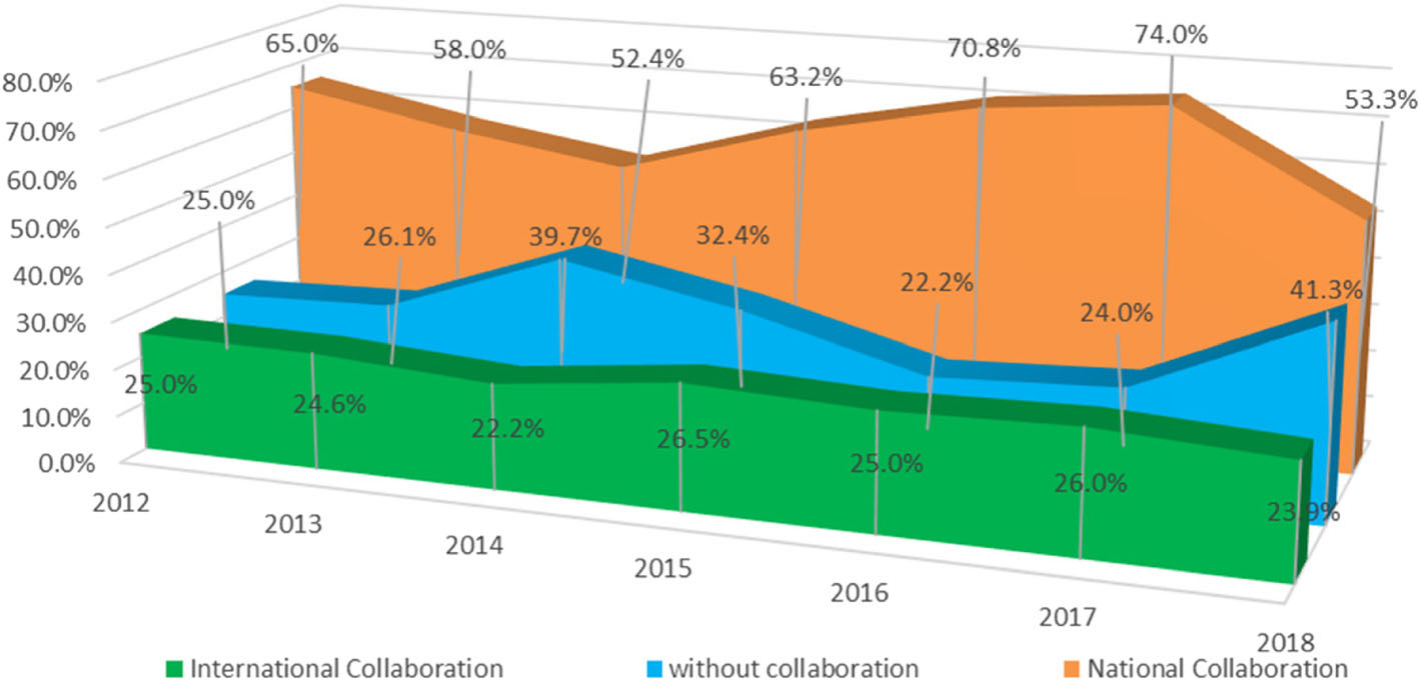
Annual evolution of collaboration between institutions (source: Scopus database)

The percentage of works carried out in domestic collaboration was 61.6%, while the percentage of works published with international collaboration was 24.7%. A total of 31.1% of the papers lacked collaboration, as a single institution signed them. Figure 4 shows the evolution of the works according to these types of collaboration. The sum of the three percentages exceeds 100% because domestic and international collaboration can coexist in the same work. The rate of collaboration for the institutions was 2.74 institutions per work.

**Fig. 5:**
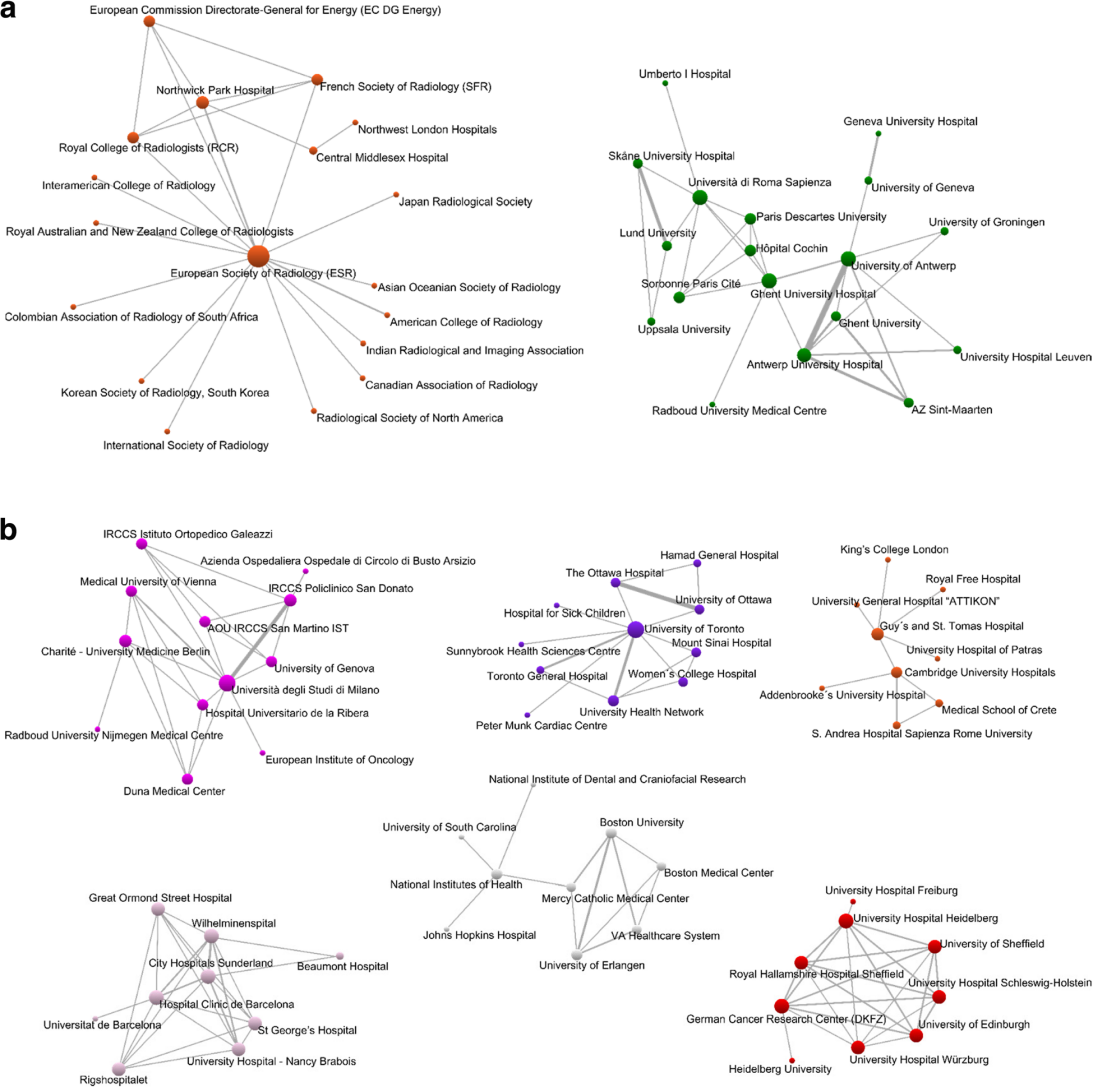
Network of collaboration between institutions. **a** Groups with 21 institutions. **b** Groups between 9 to 11 institutions (source: Scopus database)

The analysis of social networks applied to collaboration among institutions identified 38 groups whose institutions published at least one collaborative work, in which 170 different institutions participated (Fig. 5a, b). Two groups included 18 participants (Fig. 5a). The first group had the European Society of Radiology as its central institution, and most of the signatory institutions were continental or country-specific associations or colleges. The second group included institutions from several countries, such as Antwerp University Hospital, Ghent University Hospital, and Sapienza Università di Roma, among others (Fig. 5a). In Fig. 5b, six groups were drawn, one had 12 members, one had 11, and 4 had 9 members. The group with 12 institutions had as its central institution Università degli Studi di Milano, and Italian institutions predominated. The group with 11 components had as its central institution the University of Toronto, and Canadian institutions lead it. The 9-component groups comprise mainly institutions from the UK, the USA, France, Spain, and Germany.

#### Collaboration among countries

**Fig. 6:**
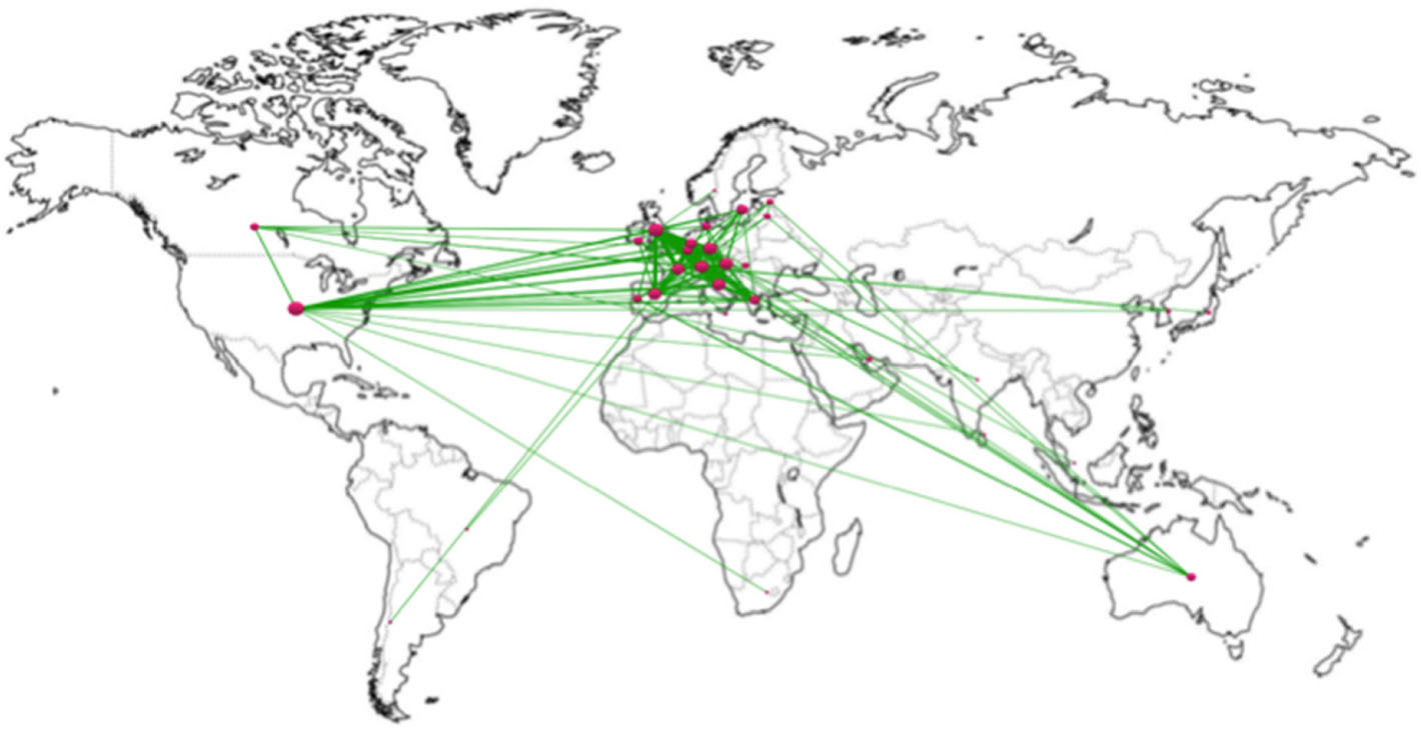
World map of collaboration between countries

Figure 6 presents the world map of collaboration among the 31 countries that have collaborated in at least one paper. The greatest number of works published in collaboration has been between the UK with Italy (*n* = 14), Austria (*n* = 12), France (*n* = 11), and Greece (*n* = 10). In addition to the striking collaboration among European countries, it is worth highlighting the collaboration between these countries and the USA and Australia.

### Content analysis

**Fig. 7:**
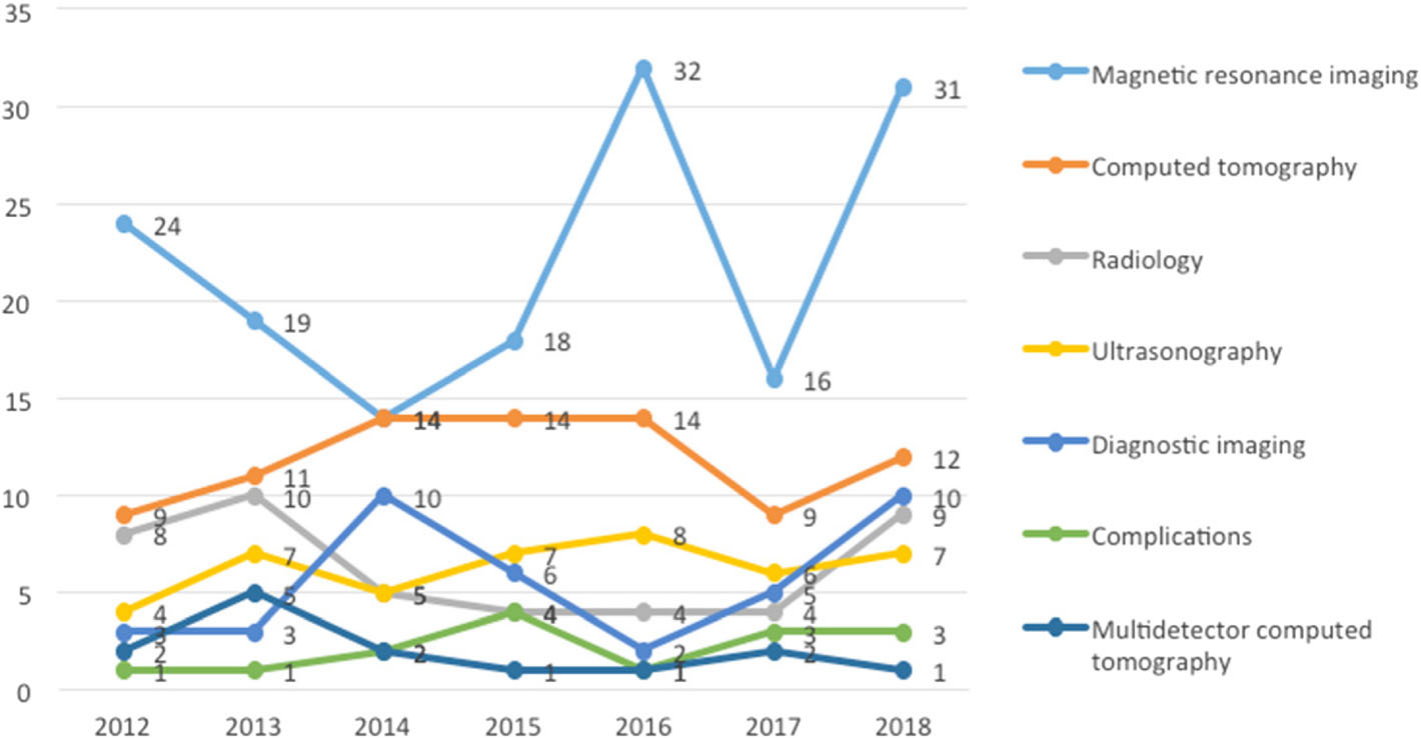
Annual evolution of 7 most frequent keywords

Table 3 shows the annual evolution of the 35 keywords with 5 or more occurrences. The most frequent words were ‘Magnetic resonance imaging’ (*n* = 154), followed by ‘Computed tomography’ (*n* = 83), ‘Ultrasonography’ (*n* = 44), ‘Radiology’ (*n* = 44), and ‘Diagnostic imaging’ (*n* = 39). Figure 7 illustrates the evolution of the 7 most frequent keywords.

**Table 3:** Most frequent keywords

Keyword	2012	2013	2014	2015	2016	2017	2018	Papers
Magnetic resonance imaging	24	19	14	18	32	16	31	154
Computed tomography	9	11	14	14	14	9	12	83
Radiology	8	10	5	4	4	4	9	44
Ultrasonography	4	7	5	7	8	6	7	44
Diagnostic imaging	3	3	10	6	2	5	10	39
Complications	1	1	2	4	1	3	3	15
Multidetector computed tomography	2	5	2	1	1	2	1	14
Breast cancer	1	2	1	1	1	3	3	12
Paediatrics	3	2	1	4	1			11
Tomography X-ray computed	3	4			1		3	11
Mammography	1	1	1	1	2	3	1	10
Radiation dose		2	2	2	1	1	1	9
Computed tomography angiography	2	1			1	2	2	8
Diffusion weighted imaging	1			1	4		2	8
Radiation protection		3	1		1	2	1	8
Magnetic resonance imaging angiography	1	2			1	1	2	7
Positron emission tomography-computed tomography	1	2	1	1	1	1		7
Teleradiology		2	1	1	2		1	7
Anatomy		3			1		2	6
Education and training	1		1			2	2	6
Lung	3		1	1	1			6
Angiography	2				2		1	5
Breast	1	1			2		1	5
Cardiac	1				2	2		5
Liver	2			1	1	1		5
Malignancy		1		2	1		1	5
Multiple detector computed tomography	2		1	1			1	5
Musculoskeletal		1		1		1	2	5
Pitfalls			2	1	1		1	5
Pulmonary embolism	3		1		1			5
Quality assurance		2				1	2	5
Radiographers		1	1		1	2		5
Spine		1		1	2		1	5
Trauma			1	1	2	1		5

**Fig. 8:**
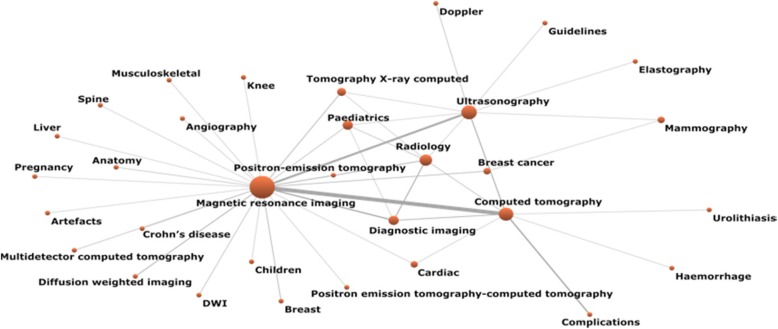
Network of co-words

The relations among the words that coincide at least twice in the set of papers (co-words) are shown in Fig. 8. In this figure, the pairs that stand out are ‘Magnetic resonance imaging’ with ‘Computed tomography’ (*n* = 32), with ‘Ultrasonography’ (*n* = 17), and with ‘Diagnostic imaging’ (*n* = 10). Another relevant relationship is in between ‘Computed tomography’ and ‘Complications’ (*n* = 11).

### Citation and impact indicators

The 22 papers having received more than 30 citations are listed in Table 4. The most frequently cited work (*n* = 260) was published in 2012 by the Davnall et al. group, affiliated to the King’s College London, Guy’s and St Thomas’ NHS Foundation Trust, and Mount Vernon Hospital (UK). Next, two papers on magnetic resonance imaging (MRI) received approximately 100 citations. The first (*n* = 111) corresponds to the group of Biederer et al., which is affiliated with the University Hospital of Schleswig-Holstein, Würzburg, and Leipzig (Germany); the second (*n* = 98) corresponds to the group of Wild et al., which belonging to the Royal Hallamshire Hospital Sheffield, University Hospital Freiburg, and Heidelberg University (Germany).

**Table 4:** Most cited papers (*n* > 30 citations)

Authors	Title	Source	Citations	Standardised citations*
Davnall F, Yip CSP, Ljungqvist G, Selmi M, Ng F, et al.	Assessment of tumor heterogeneity: an emerging imaging tool for clinical practice?	Insights into Imaging 2012; 3 (6): 573–589	260	37.14
Biederer J, Beer M, Hirsch W, Wild J, Fabel M, Puderbach M, et al.	MRI of the lung (2/3). Why... when ... how?	Insights into Imaging 2012; 3 (4): 355–371	111	15.86
Wild JM, Marshall H, Bock M, Schad LR, Jakob PM, Puderbach M, et al.	MRI of the lung (1/3): methods	Insights into Imaging 2012; 3 (4): 345–353	98	14.00
Lobbes MBI, Prevos R, Smidt, M, Tjan-Heijnen VCG, van Goethem M, Schipper R, et al.	The role of magnetic resonance imaging in assessing residual disease and pathologic complete response in breast cancer patients receiving neoadjuvant chemotherapy: a systematic review	Insights into Imaging 2013; 4 (2): 163–175	70	11.67
Ching ASC, Kuhnast B, Damont A, Roeda D, Tavitian B, Dollé F.	Current paradigm of the 18-kDa translocator protein (TSPO) as a molecular target for PET imaging in neuroinflammation and neurodegenerative diseases	Insights into Imaging 2012; 3 (1): 111–119	70	10.00
Biederer J, Mirsadraee S, Beer M, Molinari F, Hintze C, Bauman G, et al.	MRI of the lung (3/3)-current applications and future perspectives	Insights into Imaging 2012; 3 (4): 373–386	67	9.57
Khawaja AZ, Cassidy DB, Al Shakarchi J, McGrogan DG, Inston NG, Jones RG.	Revisiting the risks of MRI with Gadolinium based contrast agents—review of literature and guidelines	Insights into Imaging 2015; 6 (5): 553–558	58	14.50
Mauri G, Porazzi E, Cova L, Restelli U, Tondolo T, et al.	Intraprocedural contrast-enhanced ultrasound (CEUS) in liver percutaneous radiofrequency ablation: Clinical impact and health technology assessment	Insights into Imaging 2014; 5 (2): 209–216	53	10.60
Kristensen MS, Teoh WH, Graumann O, Laursen CB.	Ultrasonography for clinical decision-making and intervention in airway management: From the mouth to the lungs and pleurae	Insights into Imaging 2014; 5 (2): 253–279	47	9.40
Juanpere S, Cañete N, Ortuño P, Martínez S, Sanchez G, Bernado L.	A diagnostic approach to the mediastinal masses	Insights into Imaging 2013; 4 (1): 29–52	42	7.00
Geenen RWF, Kingma HJ, van der Molen AJ.	Contrast-induced nephropathy: pharmacology, pathophysiology and prevention	Insights into Imaging 2013; 4 (6): 811–820	39	6.50
Griffin N, Charles-Edwards G, Grant LA.	Magnetic resonance cholangiopancreatography: the ABC of MRCP	Insights into Imaging 2012; 3 (1): 11–21	38	5.43
Filippiadis DK, Tutton S, Mazioti A, Kelekis A.	Percutaneous image-guided ablation of bone and soft tissue tumours: a review of available techniques and protective measures	Insights into Imaging 2014; 5 (3): 339–346	37	7.40
Purohit BS, Ailianou A, Dulguerov N, Becker CD, Ratib O, Becker M.	FDG-PET/CT pitfalls in oncological head and neck imaging	Insights into Imaging 2014; 5 (5): 585–602	35	7.00
Hayashi D, Hamilton B, Guermazi A, de Villiers R, Crema MD, Roemer FW.	Traumatic injuries of thigh and calf muscles in athletes: role and clinical relevance of MR imaging and ultrasound	Insights into Imaging 2012; 3 (6): 591–601	35	5.00

*Number of citations divided by the number of years since the paper was published until 2018

A summary of the main citation indicators can be seen in Table 5. The number of citations received in Scopus (4295) exceeds the number of citations received in ESCI (1177). The average number of citations per paper was 9.06 in Scopus versus 4.03 in ESCI.

**Table 5:** Main impact indicators

Indicator	Scopus	Emerging Sources Citation Index (ESCI)
Total citations received	4295	1177
Average citations per paper	9.06	4.03
H-index	29	16
SJR in 2017	0.868	–
Ranking	74 (of 330 journals)	
Quartile in 2017	1	–
Number of papers with more than 100 citations	3	0
Number of papers with more than 50 citations	8	2

*Data on the number of citations were obtained on 19 January 2019

The SJR indicator improved in value over the years analysed, from 0.591 in 2013 to 0.868 in 2017, with an upward trend in 2016 and 2017. *Insights into Imaging* was included in 2013 and 2016 in the second quartile of the Radiology, Nuclear Medicine and Imaging area, while in 2014, 2015, and 2017, it was placed in the first quartile.

*Insights into Imaging*’s position in the Radiology, Nuclear Medicine and Imaging world ranking has also improved in recent years, from 109 (out of 305 journals) in 2013 to 74 (out of 330 journals) in 2017.

The h-index *of Insights into Imaging* in Scopus was 29, which places the journal in 154th place in the world ranking of the Radiology area (2017). Although this value is not very high, one should keep in mind that the journal’s youth prevents this indicator from being higher. The h-index was 16 in ESCI.

## Discussion

This scientometric research focused on the analysis of the journal *Insights into Imaging* to quantify its coverage in Scopus and ESCI and the evolution of its most relevant bibliometric indicators. The calculation of the main bibliometric indicators and the analysis of social networks necessary to identify the groups of authors and institutions, as well as the collaboration among countries and the subject-based study obtained from keywords, have been carried out on the basis of the papers indexed in Scopus. We have not chosen the ESCI database from the Web of Science as the main source of data because it only included 292 records, compared to the 474 indexed in Scopus; however, some data from ESCI have been taken into account in order to compare them with those obtained in Scopus. The coverage of the journal in these databases does not exactly coincide, both in the number of annual articles and in the documentary typology of the articles included. For example, in Scopus, the journal is indexed from 2012 to 2018, while it has been indexed in ESCI on a regular basis since 2015. These differences in index coverage create discrepancies in bibliometric indicators, depending on whether they are extracted from one or the other database.

As we have seen, numerous authors participated in the publication of studies and belong to numerous institutions and countries around the world, although what is striking is the leadership of Italian institutions and the participation of European countries in almost all of those published works. This is probably because it is the official journal of the European Society of Radiology (https://insightsimaging.springeropen.com/about).

The percentage of editorial board members who have published in the journal was 46.9%, a value somewhat lower than that found in other areas such as addiction, in which it was 52.1% [19]. The percentage of papers published by these members was 11.8%, which is slightly higher than that found in other areas such as agriculture (7.7%) [20], information sciences (8%) [21], and various other medical subspecialties (7.7%) [22]. However, one should kept in mind that these studies included a high percentage of original articles, while those included in *Insights into Imaging* are fundamentally revisions.

Social network analysis has made it possible to identify those groups with the most active collaboration in the field and that can be considered at the forefront of research in the area. As for collaboration among countries, the collaboration among European countries once again predominates, to which must be added the collaboration among Europe and the USA and Australia, in particular.

Keyword analysis has shown the predominance of studies with technologies, such as ‘Magnetic resonance imaging’, ‘Computed tomography’, ‘Radiology’, and ‘Ultrasonography’. Within the group of diseases, ‘Breast cancer’ and ‘Paediatrics’ are highlighted. In another outstanding group of works, topics such as ‘Complication’, ‘Radiation dose’, and ‘Radiation protection’ are studied. Notable keywords include education and training, a topic to which 6 papers have been devoted, some of them published by the European Society of Radiology, which is an example of the interest of this society in topics related to formation and training in the area [23, 24].

Citation analysis provides metrics that allow us to observe the impact of authors and their institutions on their respective fields, as well as highly influential studies [7, 9, 25]. The average citation amount per paper in Scopus was more than twice as high as in ESCI, which is logical since Scopus includes papers from 2010 (and ESCI only from 2015); thus, earlier papers have had a greater chance to be cited. Another reason for the higher citation amount in Scopus is the greater coverage of Scopus journals. In Radiology, Nuclear Medicine and Imaging, Scopus indexes 330 journals, to which we can add the 55 included in the Radiological and Ultrasound Technology area, while the Web of Science Core Collection only includes 129 in the corresponding area of Radiology, Nuclear Medicine and Medical Imaging.

In 2017, *Insights into Imaging* was placed in the first quartile of the subject category Radiology, Nuclear Medicine and Medical Imaging of the SJR. If we consider the 127 journals included in this category, both in JCR and in SJR and ordered by the value of the SJR, *Insights into Imaging* would be placed in the second quartile of the JCR. On the other hand, the average citation/work in *Insights into Imaging* in ESCI is 4.03, while the average of the other journals in this area included in ESCI is 1.43. If only reviews are taken into account, the citation/work indicator in *Insights into Imaging* is 4.49, while in the other journals, it is 3.03. The data provided on citations validate that *Insights into Imaging* is considered one of the main vehicular open journals of imaging diagnosis worldwide. All these data support the transfer of *Insights into Imaging* from ESCI to the Science Citation Index Expanded.

As with the number of citations received, the journal’s youth and the number of years it has been indexed in citation databases also influence the value of the h-index. Although in 2017 it was in the middle of the ranking of journals in the field of radiology (number 154 out of 305), it is to be assumed that this position will improve in the coming years, as it continues to be published and consolidated in its area.

The most frequently cited articles in *Insights into Imaging* provide an overview of the main developments and allow us to recognise the important advances in this field, since the articles frequently cited by inference imply that they are the most read and are thus of major importance within that specialty. The themes of these most cited works are diverse, and it is noteworthy that 9 of the most cited works have MRI as their central theme.

### Limitations

All bibliometric studies have limitations that should be discussed. First, there is a possible bias in the coverage of the main source used in this work (Scopus), since it is possible that it has not picked up some studies published in the journal or that some work is duplicated [26]. However, the databases considered in this work enjoy significant international prestige and provide detailed citation metrics; they are thus the most frequently used in bibliometric studies and are considered today as the gold standard of these studies [26]. Second, it is possible that inaccuracies in standardisation may have affected the quantification of the production of certain authors or institutions, or the classification of works according to keywords. However, a rigorous process of standardisation of the names of authors, institutions, and keywords has been carried out. It is also important to note that citation analysis is not necessarily a measure of study quality and is not the only methodology for assessing the impact of research. Lastly, some quality work may not have been cited because not enough time has elapsed since its publication.

### Future work

As future work, it would be interesting to observe the evolution of the identified groups of co-authors and institutions, as well as the evolution of the indicators of the journal’s production, collaboration, and impact. This information could be helpful in taking strategic decisions aimed at correcting the detected weaknesses or in continuing along the path of the journal’s strengths. It would also be useful to analyse the citation flows from the journal to other journals and from these to *Insights into Imaging*.

## Data Availability

The datasets used and/or analysed during the current study are available from the corresponding author on reasonable request.

## Abbreviations

ESCI: Emerging Sources Citation Index
ESR: European Society of Radiology
MRI: Magnetic resonance imaging
SJR: SCImago Journal Rank

## References

[1] Martí-Bonmati L (2018). Letter from the new editor-in-chief for insights into imaging. Insights Imaging.

[2] Miguel-Dasit Alberto, Martí-Bonmatí Luis, Aleixandre Rafael, Sanfeliu Pilar, Valderrama Juan C. (2004). Spanish Production of Research Articles on Diagnostic Imaging in Cardiology and Radiology (1994-1998). Revista Española de Cardiología (English Edition).

[3] Miguel Dasit A, Martí-Bonmatí L, Aleixandre R, Sanfeliu P, Valderrama JC (2005). Análisis comparativo de la producción española sobre diagnóstico por ecografía en las especialidades de obstetricia y ginecología y radiodiagnóstico (1994-1998). Radiología.

[4] Miguel-Dasit Alberto, Aleixandre Rafael, Valderrama Juan C., Martí-Bonmatí Luis, Sanfeliu Pilar (2005). Hypothetical influence of non-indexed Spanish journals on the impact factor of radiological journals. European Journal of Radiology.

[5] Miguel-Dasit A, Martí-Bonmatí L, Sanfeliu P, Aleixandre R (2007). Cardiac MR imaging: balanced publications by radiologists and cardiologists. Radiology.

[6] Marwick Thomas H., Chandrashekhar Y., Achenbach Stephan, Dilsizian Vasken, Fayad Zahi A., Finn Aloke V., Hundley W. Gregory, Kern Morton J., Kramer Christopher M., Sengupta Partho P., Shaw Leslee J., Zoghbi William A., Narula Jagat (2011). Bibliographic Metrics at JACC: Cardiovascular Imaging. JACC: Cardiovascular Imaging.

[7] Choudhri AF, Castillo M (2015). Subspecialty virtual impact factors within a dedicated neuroimaging journal. AJNR Am J Neuroradiol.

[8] Khan MS, Ullah W, Riaz IB (2016). Top 100 cited articles in cardiovascular magnetic resonance: a bibliometric analysis. J Cardiovasc Magn Reson.

[9] Mohammed Mohammed Fahim, Chahal Tejbir, Gong Bo, Bhulani Nizar, O’Keefe Michael, O’Connell Timothy, Nicolaou Savvas, Khosa Faisal (2017). Trends in CT colonography: bibliometric analysis of the 100 most-cited articles. The British Journal of Radiology.

[10] Zhai X, Cui J, Shao J (2017). Global research trends in spinal ultrasound: a systematic bibliometric analysis. BMJ Open.

[11] Yao X, Yan J, Ginda M, Börner K, Saykin AJ, Shen L (2017). Alzheimer’s disease neuroimaging initiative. Mapping longitudinal scientific progress, collaboration and impact of the Alzheimer’s disease neuroimaging initiative. PLoS One.

[12] Aleixandre-Benavent Rafael, Zurián Juan Carlos Valderrama, Miguel-Dasit Alberto, Arroyo Adolfo Alonso, Gómez Miguel Castellano (2007). Hypothetical influence of non-indexed Spanish medical journals on the impact factor of the Journal Citation Reports-indexed journals. Scientometrics.

[13] Tijssen RJ, Visser MS, Van Leeuwen TN (2002). Benchmarking international scientific excellence: are highly cited research papers an appropriate frame of reference?. Scientometrics.

[14] Hirsch J. E. (2005). An index to quantify an individual's scientific research output. Proceedings of the National Academy of Sciences.

[15] Durieux V, Gevenois PA (2010). Bibliometric indicators: quality measurements of scientific publication. Radiology.

[16] Albarrán Pedro, Ortuño Ignacio, Ruiz-Castillo Javier (2011). High- and low-impact citation measures: Empirical applications. Journal of Informetrics.

[17] Bornmann L (2014). How are excellent (highly cited) papers defined in bibliometrics? A quantitative analysis of the literature. Res Eval.

[18] Waltman Ludo (2016). A review of the literature on citation impact indicators. Journal of Informetrics.

[19] Vidal-Infer A (2010). Análisis de los artículos originales publicados en revistas especificas sobre drogodependencias incluidas en el Journal Citation Reports (2002–2006) (doctoral theses).

[20] Zdenek R, Lososova J (2018). An analysis of editorial board members’ publication output in agricultural economics and policy journals. Scientometrics.

[21] Walters WH (2015). Do editorial board members in library and information science publish disproportionately in the journals for which they serve as board members?. J Sch Publ.

[22] Luty J, Arokiadass SMR, Easow JM, Anapreddy JR (2009). Preferential publication of editorial board members in medical specialty journals. J Med Ethics.

[23] European Society of Radiology (ESR) (2015) Research education in Europe: an opinion paper by the European Society of Radiology. Insights Imaging 6(2):157–162 10.1007/s13244-015-0397-x PMC4376819 25763995

[24] European Society of Radiology (ESR) (2018) Radiology trainees forum survey report on workplace satisfaction, ESR education, mobility and stress level. Insights Imaging 9(5):755–759 10.1007/s13244-018-0649-7 PMC6206381 30187269

[25] Gonzalez de Dios J, Alonso Arroyo A, Aleixandre-Benavent R (2019). Half a century of Anales de Pediatría. Evolution of its main bibliometric indicators in the Web of Science and Scopus international databases. An Pediatr (Barc).

[26] Valderrama JC, Aguilar-Moya R, Melero-Fuentes D, Aleixandre-Benavent R (2015) A systematic analysis of duplicate records in Scopus. J Infometr 9:570–576

